# Effect of intramuscular injection of human chorionic gonadotropin on endometrium preparation in frozen-thawed embryo transfer cycle: A randomized clinical trial

**DOI:** 10.22088/cjim.14.2.185

**Published:** 2023

**Authors:** Firoozeh Akbari Asbagh, Fatemeh Ghasemzadeh, Mahbod Ebrahimi, Fatemeh Davari-Tanha, Elham Feizabad, Parvin Akbari Asbagh, Samaneh Hosseini Quchani

**Affiliations:** 1 Department of Infertility, Yas Hospital, Tehran University of Medical Sciences, Tehran, Iran; 2 Vali-E-Asr Reproductive Health Research Center, Family Health Research Institute, Tehran University of Medical Sciences, Tehran, Iran; 3 Department of Pediatrics, School of Medicine, Imam Khomeini Hospital, Tehran University of Medical Sciences, Tehran, Iran

**Keywords:** In vitro fertilization, Human chorionic gonadotropin, Pregnancy outcome

## Abstract

**Background:**

Assisted reproductive therapy (ART) has been developed remarkably in these decades; however, the rate of unsuccessful embryo implantation especially in the frozen-thawed embryo transfer (FET) cycles remains high and is reported up to 70%. The current study was designed to compare the effect of intramuscular injection of hCG on endometrium preparation and embryo implantation, in women undergoing FET compared to the control group.

**Methods:**

This clinical trial was done on 140 infertile women that underwent FET. The study sample was randomly allocated to the intervention group (two 5000 unit ampoules of hCG were injected intramuscularly before the first dose of progesterone administration) and the control group (without hCG injection). In both groups, 4 days after progesterone administration, the cleavage stage embryos were transferred. The study outcomes were biochemical pregnancy, clinical pregnancy and abortion rate.

**Results:**

The average age of intervention and control group was 32.65±6.05 and 33.11±5.36 years, respectively. The basic information between two study groups did not differ significantly. The chemical (30% vs. 17.1%, P=0.073, relative risk (RR)=0.57) and clinical (28.6% vs. 14.3%, P=0.039, RR=0.50) pregnancy rates were higher in the intervention group compared to the control group; these higher ratios were only significant in clinical pregnancy rate. Abortion rate was not significantly (P=0.620) different between the intervention and control groups (4.3% vs. 1.4%, respectively).

**Conclusion:**

This study showed that intramuscular injection of 10000 IU hCG before the endometrial secretory transformation phase in cleavage-stage embryo, improves IVF cycle outcomes.

Assisted reproductive therapy (ART) has been developed remarkably in these decades; however, the rate of unsuccessful embryo implantation especially in the frozen-thawed embryo transfer (FET) cycles remains high and is reported up to 70% (1). The implantation process is highly complex requiring appropriate interface between endometrium and embryo (2). In this regard, some maternal and fetal factors including maternal age, hormonal level rising, quality of embryos, as well as endometrial embryo acceptance have important roles (3, 4). Preparing an endometrium for implantation in FET cycles has been done with the prescription of sequential estradiol and progesterone supplementation.

However, the luteinizing hormone (LH) surge which happens at the luteal-phase of a natural cycle has not been achieved in this artificial cycle (5). Evidence showed that human chorionic gonadotropin (hCG), which its receptor is similar to LH receptor, can be a proper alternative to induce the mechanisms run by LH surge in a natural cycle (6-9). In fact, hCG mimics LH roles by adhering to LH/hCG receptors which is located in the endometrium and ovaries, then induces endometrial maturation, decidualization, angiogenesis, synchrony promotion, oocyte and corpus luteum maturation, cytotrophoblast proliferation and invasion, maternal immune system regulation, and uterine contraction suppression (2).

While former studies evaluated the effect of injection of hCG on IVF cycle outcomes, their results are controversial and not suggested as a routine intervention in practice, yet. Hence, the current study was designed to compare the effect of intramuscular injection of hCG on endometrium preparation and embryo implantation, in women undergoing FET compared to the control group.

## Methods

This randomized clinical study was done on 140 infertile women referred to our IVF unit and were candidates for FET cycle, at Yas hospital, Tehran, Iran from December 2021 to June 2022. This study was approved by the Ethics Committee of Tehran University of Medical Sciences (IR.TUMS.MEDICINE.REC.1400.891). The study was registered in the Iranian randomized clinical trial registry website (IRCT20211121053124N1) and was done in compliance with Helsinki declaration. 

The informed consent forms were signed with all the participants before they enrolled the study. The study population consisted of infertile women with female infertility that caused them to undergo FET cycle, and having at least one grade A or B embryo. Women with body mass index (BMI) greater than 30 kg/m^2^, had uterine anomalies, high-grade endometriosis, hydrosalpinx (if not removed or ligated by surgery), endometrial thickness (ET) less than 7.5 mm, oocyte donor, as well as withdrawal from participation, were excluded.

The corresponding author generated the random allocation sequence, enrolled participants and randomly assigned 70 participants in the intervention group (received 10000 units hCG) and 70 in the control group using the random allocation rule. Random allocation rule was applied as randomization method: First, 70 letters A and 70 letters B were written on small papers. Then all of them were placed in a bag and for each patient, after obtaining informed consent, a paper was selected randomly and without replacement, and based on the letter written on it, the desired intervention was performed for the patient. In addition, interventions A (intramuscular injection of human chorionic gonadotropin) or B (control group) are determined by a lottery. Since the nature of intervention (intramuscular injection), and not placebo using, the patients known their group allocation. In addition, the correspondent author that allocated the group and performed the fetus transferring, was not blind. Only the outcome assessor and the analyzer (not knowing about the treatment group codes in the SPSS data sheet) did not know the type of treatment. 

For all participants, FET was scheduled. On the second or third day of the mensuration cycle, 4 mg per day of estradiol valerate (Abu Reihan Pharmaceutical Company, Iran) was initiated and continued for four days, followed by 6 mg per day of estradiol for the next four days. Then, on the eighth day, serial vaginal ultrasound (Honda Company, Japan) was performed to check ET. When ET reached ≥ 7.5 mm and a three-line view of the endometrium was observed, in the intervention group, 10000 units of hCG (two 5000-unit ampoules, Puyesh Pharmaceutical Company) were injected intramuscularly before the first dose of progesterone administration. The control group did not receive any hCG.

In both groups, 100 mg per day of intramuscular progesterone (Iran Hormone Company, Iran) combined with estradiol valerate were administered for 4 days. On day 4 after progesterone, the cleavage stage embryos were transferred. The following data were recorded for both groups: age, marriage and infertility duration, infertility cause, BMI, fasting blood sugar (FBS), thyroid-stimulating hormone (TSH), follicle-stimulating hormone (FSH), anti-mullerian hormone (AMH), ET, and embryo characteristics. The study outcomes were biochemical pregnancy (serum β-hCG ≥ 10 Iu/L on the fourteenth day after embryo transfer), clinical pregnancy (gestational sac observation by TVS at 5 to 6 weeks of gestation), and positive FHR (embryo heart rate detection by TVS at 6 to 7 weeks of gestation) and abortion rate (pregnancy loss under 20 weeks of gestation).

All of the statistical analyses were done using SPSS version 24.0. P-values less than 0.05 were considered statistically significant. Independent t-test and non-parametric Mann-Whitney U test were used to evaluate the differences in means. A chi-square test and Fisher's exact test were applied to assess the differences in proportions.

## Results

One hundred and forty-six infertile women were evaluated for eligibility; of these, 6 women were excluded due to: severe azoospermia in their partners (n = 2), leiomyoma (n = 1), and declined to participate (n = 3).

**Figure 1:**
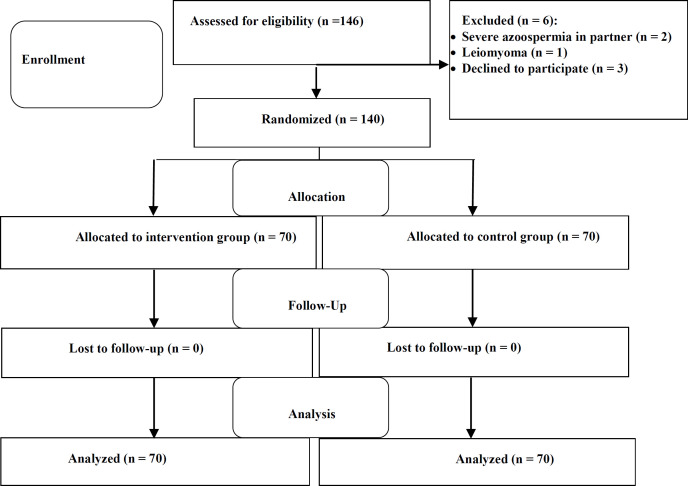
Consort flow diagram of the infertile women

 In total, 140 women were randomized to the study groups and were analyzed (figure 1). The average age was 32.65±6.05 and 33.11±5.36 years in the intervention and control group, respectively with non-significant (P=0.637) difference. Furthermore, BMI was 25.21±2.11 and 25.86±2.08 kg/m^2^ on average, in the intervention and control group, respectively (P=0.069). The basic information of participants did not vary significantly between the two study groups (table 1). 

 In addition, the two study groups were statistically similar as regards to infertility duration, the prevalence of irregular menstruation, polycystic ovarian syndrome (PCOS), unexplained infertility (UEI), diminished ovarian reserve (DOR), low-grade endometriosis, and tubal factor (TF), as well as previous FET number (table 1, 2). The mean number of transferred embryos was 2.17±0.61 in the intervention group and 2.18±0.62 in the control group without any significant (P=0.089) difference between the two study groups.

In our IVF center in both study groups, positive ß-hCG was observed in 33 women (47.1%), and clinical pregnancy in 30 (42.9%) women. 

The chemical (30% vs. 17.1%, P=0.073, relative risk (RR) =0.57) and clinical (28.6% vs. 14.3%, P=0.039, RR=0.50) pregnancy rates were higher in the intervention group compared to the control group; although these higher ratios were only significant regarding clinical pregnancy rate (table 3). Abortion rate was not significantly (P=0.620) different between the intervention and control groups (4.3% vs. 1.4%, respectively). No case of ectopic pregnancy and multiple pregnancies were detected in the two study groups (table 3).

**Table 1:** The basic characteristics of participants

**Variables**	**Control**	**Intervention**	**P-value**
**Partner age (Y)**	36.17±4.46	36.88±5.75	0.414
**Age (Y)**	33.11±5.36	32.65±6.05	0.637
**Duration of Marriage (Y)**	8.06±4.07	8.59±5.14	0.501
**BMI (kg/m** ^2^ **)**	25.86±2.08	25.21±2.11	0.069
**FBS (mg/dL)**	87.51±7.28	89.02±6.35	0.192
**TSH (mIU/L)**	2.23±0.88	2.06±0.97	0.289
**FSH (mIU/mL)**	6.30±2.51	6.77±2.58	0.281
**Primary infertility duration (Y)**	5.07±4.15	4.50±4.52	0.443
**Secondary infertility duration (Y)**	1.03±2.01	1.54±2.7	0.211
**AFC (n)**	10.8±4.32	9.72±4.16	0.138
**AMH (ng/ml)**	4.05±3.65	4.11±3.68	0.920
**Endometrial thickness (mm)**	8.18±0.55	8.11±0.54	0.450
**Transfer number**	1.77±0.99	1.5±0.83	0.082
**IVF** **number**	1.42±0.71	1.24±0.49	0.076
**Transfer fetus** **number**	2.18±0.62	2.17±0.61	0.891

BMI: body mass index, FBS: fasting blood sugar, TSH: thyroid-stimulating hormone, FSH: follicle-stimulating hormone, AFC: antral follicle count, AMH: anti-mullerian hormone, IVF: in vitro fertilization

**Table 2:** Comparison of cycle characteristics in the two study groups

**Variables**	**Study groups**		**P-value**
	**control**	**Intervention**	
**Irregular ** **menstruation**	14 (20)	23 (32.9)	0.125
**Polycystic ovary syndrome**	27 (38.6)	25 (35.7)	0.861
**Unexplained infertility**	24 (34.3)	22 (31.4)	0.857
**Diminished ovarian reserve**	15 (21.4)	15 (21.4)	1.000
**Low-grade endometriosis**	0	5 (7.1)	0.058
**Tubal factor**	7 (10)	5 (7.1)	0.764
**A**	39 (55.7)	41 (58.6)	0.864
**B**	55 (78.6)	53 (75.7)	0.841
**C**	30 (42.9)	30 (42.9)	1.000

**Table 3:** The frequency of the study outcomes in the intervention and control groups

**Variables**	**Study group**		**Relativerisk (Confidence interval 95%)**	
	**Control**	**Intervention**		**P-value**
**Chemical pregnancy**	12 (17.1)	21 (30)	0.57 (0.30-1.07)	0.073
**Clinical pregnancy**	10 (14.3)	20 (28.6)	0.50 (0.25-0.99)	0.039
**Positive FHR **	10 (14.3)	20 (28.6)	0.50 (0.25-0.99)	0.039
**Abortion**	1 (1.4)	3 (4.3)	0.33 (0.36-3.12)	0.620

FHR: fetal heart rate

## Discussion

The hCG has positive effects on endometrial receptivity and embryo implantation through different cellular and molecular pathways. Although, the effect of intrauterine hCG injection has been reported in several previous studies (10-14), this study showed higher pregnancy rate with IM injection of hCG before the endometrial secretory transformation phase in artificially prepared FET cycle compared to the control group. In the line of our findings, Ling Deng et al. 2020’s study (2), evaluated the effects of IM hCG injection which supported the positive effect of exogenous hCG injection; although, in blastocyst stage embryo hCG injection did not have significant effects. The positive effect of IM hCG administration, also were reported in Jan Tesarik et al. ‘s(2003)study (15) which was done in oocyte recipients who underwent GnRH agonist pretreatment down regulation, in Robab Davar et al.’s (2016) study (16) which was done in infertile women with at least 2 previous failed IVF cycle and thin endometrium (thickness <7 mm), and in Yanbo Du et al. ‘s(2016)study (17) which was done in women with endometriosis-associated infertility. It is worth to mention that even with lower doses of IM hCG administrations, its benefits on IVF outcomes were detected; for instance, in Robab Davar et al.’s (2016) study (16), the hCG positive effect reached with 150 IU hCG administration from the 8th day of cycle until ET was at least 7 mm and in Yanbo Du et al.’s (2016) study (17), with 8000 IU hCG injection before progestin administration.

In contrast to our study, in Eftekhar et al.’s study (18) no statistically significant differences were detected regarding IVF outcomes in hCG group compare with the control group. In that study, intervention group received hCG twice, during their FET cycles (5000 IU on the day of progesterone prescription and 5000 IU on the day of embryo transfer).

In addition, positive effects of intrauterine hCG infusion and higher pregnancy rates were reported in several studies (4, 19-21) while in other (22-24) significant correlations were not detected. It may be due to its adverse effects including uterine contraction promotion, endometrium scratching or bleeding, and excessive uterine fluid can displace and expulse the embryo and consequently the pregnancy losses.

More investigation to increase the successful implantation, especially in FET cycles and in the blastocyst embryo transfer cycles, is forced needed. Future studies are suggested to assess the effect of hCG injection in different populations and study groups (for example intrauterine injection of hCG, as a control group and IM injection of hCG, as an interventional group), as well as assessing different roots, dosage, and time of hCG injection. This study showed that intramuscular injection of 10000 IU hCG before the endometrial secretory transformation phase in cleavage-stage embryo transfer improves IVF cycle outcomes. The strength of this study was its prospective nature and its design. However, this study had some biases and limitations including the lack of using placebo, the small sample size, its low external generalization since it was done only in one center, only in the cleavage stage embryo transfer and in younger patients.
